# The P2Y_13_ Met-158-Thr Polymorphism, Which Is in Linkage Disequilibrium with the P2Y_12_ Locus, Is Not Associated with Acute Myocardial Infarction

**DOI:** 10.1371/journal.pone.0001462

**Published:** 2008-01-23

**Authors:** Stefan Amisten, Oscar Ö. Braun, Lovisa Johansson, Martin Ridderstråle, Olle Melander, David Erlinge

**Affiliations:** 1 Department of Cardiology, Lund University Hospital, Lund, Sweden; 2 Department of Clinical Sciences, Lund University, Malmö, Sweden; Innsbruck Medical University, Austria

## Abstract

**Background and Aims:**

The aims of this study were to investigate (1) if P2Y_12_ polymorphisms defining the P2Y_12_ H2 allele are associated with any other SNPs that may explain the previously reported association with increased ADP induced platelet activation and association with peripheral arterial disease and coronary artery disease and (2) if such variants are associated with acute myocardial infarction (AMI) or classical risk factors for AMI.

**Methods and Results:**

The P2Y_13_ Met-158-Thr polymorphism was found to be in linkage disequilibrium (LD) with the P2Y_12_ H2 haplotype (all examined SNPs: D′ = 1.0, r^2^ = 0.936–1.0), defining a novel P2Y_12_ H2/P2Y_13_ Thr-158 haplotype. Genotyping of an AMI case control population (n = 1244 cases, 2488 controls) revealed no association of the P2Y_13_ Thr-158 allele with AMI (OR = 0.96, 95% C.I. 0.82–1.12, P = 0.63). Also, no differences between the genotype frequencies of P2Y_13_ Met-158-Met and Met-158-Thr/Thr-158-Thr were seen in AMI case-control subpopulations (early onset AMI OR = 1.06, 95% C.I. 0.85–1.31, P = 0.62); family history of AMI (OR = 0.98, 95% C.I. 0.78–1.22, P = 0.83) nor in early onset AMIs with family history of AMI (OR = 1.0, 95% C.I. 0.74–1.36, P = 1.0). Genotyping of the P2Y_13_ Met-158-Thr polymorphism in a population based sample (n = 6055) revealed no association with cardiovascular risk factors. In addition, the P2Y_13_ Met-158-Thr polymorphism was genotyped in a diabetes case-control population, and associations were found neither with DM nor with any examined DM risk factors.

**Conclusion Genotyping:**

The P2Y_13_ Met-158-Thr polymorphism is in tight LD with the P2Y_12_ locus but is not associated with AMI or classical cardiovascular risk factors.

## Introduction

Three human adenosine diphosphate (ADP) receptors have been cloned: P2Y_1_, P2Y_12_ and P2Y_13_ [1]–[4]. On platelets, P2Y_1_ and P2Y_12_ mediate ADP-induced platelet activation and aggregation [5]. In red blood cells, activation of P2Y_13_ by the adenosine triphosphate (ATP) metabolite ADP activates a negative feedback loop that inhibits ATP release from erythrocytes [6]. Rare mutations in the P2Y_12_ gene that disrupt P2Y_12_ receptor function result in compromised ADP-induced platelet activation and increased bleeding times [1], [7], [8].

The clinical importance of the P2Y_12_ receptor as a mediator of platelet activation has become evident in several large-scale clinical studies and inhibition of the P2Y_12_ receptor with clopidogrel is one of the cornerstones in treatment and prevention of acute coronary syndromes [9]. Even greater P2Y12 inhibition by prasugrel was recently shown to be even more effective in preventing ischemic events than the standard regimen of clopidogrel [10].

A group of single nucleotide polymorphisms (SNPs) in the P2Y_12_ gene, forming the so called P2Y_12_ H2 haplotype [11], have been associated with increased platelet responsiveness to ADP and increased risk of peripheral arterial disease (PAD) [11]–[13]. Recently, Cavallari *et al* showed an association of the P2Y12 H2 haplotype with coronary artery disease (CAD) [14]. It has also been proposed that this haplotype may account for variations in response to clopidogrel. However, several studies have failed to confirm any association between platelet function and the H2 haplotype [15]–[17].

The group of polymorphisms that make up the P2Y_12_ H2 haplotype are all synonymous polymorphisms that do not change the amino acid sequence of the P2Y_12_ protein, and no mechanistic explanation to the reported increased platelet reactivity to ADP associated with this haplotype has been presented [11]. However, a non-synonymous polymorphism, Met-158-Thr, in the neighboring P2Y_13_ gene, located only 8 kb away from P2Y_12_, could be in linkage disequilibrium with the P2Y_12_ H2 haplotype. We hypothesized that the P2Y_13_ Met-158-Thr polymorphism of the P2Y_13_ receptor could account for the reported effects of the P2Y_12_ H2 haplotype since the receptors share the same ligand, ADP. The P2Y_13_ receptor has been found on red blood cells and inflammatory cells, both cell types known to interact with platelets [4], [6]. The first objective of this study was to examine possible linkage disequilibrium (LD) between the P2Y_12_ H2 haplotype and the P2Y_13_ Met-158-Thr polymorphism. After showing that this was the case, we aimed at investigating if the P2Y_13_ Met-158-Thr polymorphism is associated with acute myocardial infarction (AMI) or diabetes mellitus, two diseases strongly associated with peripheral arterial disease [18], [19]. Our hypothesis that the P2Y_12_ H2 haplotype and SNPs in LD with the P2Y_12_ H2 haplotype would be associated with AMI was strengthened further by a report linking the P2Y_12_ H2 haplotype with coronary artery disease (CAD) [14], since myocardial infarction is the major complication of CAD [20]. In order to do so, the Met-158-Thr polymorphism was genotyped in more than 10,000 individuals divided in three study populations: two sub-populations of the Malmö Diet and Cancer study (an AMI case-control population and a large population with cardiovascular risk factor data [21], [22]) and a diabetes mellitus case-control population with data on several DM risk factors [23].

## Materials and Methods

### Malmö diet and cancer population (MDC)

The study population is made up of 28098 randomly selected men (born 1923–1945) and women (born 1923–1950) living in the Swedish city of Malmö (population 250 000). Overall participation rate in the study was 41%.

A baseline examination was performed between 1991–1996, including assessment of dietary habits, a questionnaire on socio-economic, demographic and lifestyle factors, heredity, medication and previous and current diseases. Blood samples were taken and DNA, lymphocytes, granulocytes, erythrocytes and plasma/serum were stored in a biological bank [21], [22].

### AMI Case control population

On 31 December 2000 the study population was matched with the Swedish National Board of Health and Welfare's National Patient Registry and Cause of Death Registry. AMI cases (first AMI) were identified using the diagnosis criteria defined by the International Classification of Diseases, 9^th^ and 10^th^ and Revision, Clinical Modification (ICD 9 and 10); ICD 9 codes 410 in the Swedish Patient Registry or 410-414 in the Swedish Cause of death Registry; ICD 10 codes I21 in the Swedish Patient Registry and I21-I25 in the Swedish Cause of Death Registry.

Two gender- and age (±1 year) -matched AMI-free controls from the MDC population were assigned to each AMI case, resulting in a case-control material consisting of 1244 AMI cases and 2488 AMI-free controls. The myocardial infarction group was then further subdivided into early onset AMIs (EO, *n* = 622), age at first AMI event <62.8 years (median age of all first event AMI cases) and late onset AMIs (LO, *n* = 622, age at first AMI event >62.8 years). Family history AMIs (FH, *n* = 611) were defined as AMI cases where at least one blood related first degree family member had suffered an AMI, and non familial AMIs (*n* = 633) as cases without any first degree family history of AMI. 319 cases had both early onset and family history AMI (table 1). DNA was available from all cases and controls (*n* = 3732).

**Table 1:** Baseline characteristics of the AMI case control population and cardiovascular group population used for genotyping of the P2Y_13_ Thr-158 polymorphism.

	Controls (n = 2488)	All AMI cases (n = 1244)	Early onset AMI* (n = 622)	Family history AMI* (n = 611)	Early onset and Family history AMI* (n = 319)	Cardiovascular group (n = 6055)
Age (years)	62.5±6.5	62.3±6.5	59.0±6.3	62.5±6.5	58.9±6.3	57.5±5.9
Sex (% male)	74	74	79	69	75	42
Systolic blood pressure (mmHg)	147±20	150±21	145±19	150±20	145±19	141±19
Diastolic blood pressure (mmHg)	87.4±10	88.5±10	87.3±10	87.9±10	86.8±10	87.0±9.5
Body Mass Index (kg/m^2^)	26.1±3.7	26.9±4.0	26.9±3.9	27.0±4.0	26.9±4.0	25.9±4.0
Current smokers (%)	27	34	38	32	37	28

The 1244 AMI cases can be subdivided into three AMI subgroups: early onset AMI, defined as AMI occurring earlier than median age for all AMI cases, family history AMI, where at least one blood related family member also has suffered a myocardial infarction and early onset AMI with family history of AMI. Data of known cardiovascular risk factors was collected in the cardiovascular group only. * subgroups of all AMI cases.

### Cardiovascular group population

Of the MDC, 6103 individuals were randomly selected into a “Cardiovascular cohort” (MDC-CV), a sample thus being representative of MDC, in whom cardiovascular risk factors were measured, including systolic blood pressure, smoking status and anthropometric data and, in the majority (n = 5540), fasting plasma analyses of glucose, lipids and C-reactive protein (CRP). DNA for genotyping was obtained from 6055 of the 6101 selected individuals.

### Diabetes mellitus case-control population

The diabetes mellitus case control material has been described elsewhere [23]. Briefly, all study subjects originate from the Botnia region in Western Finland and the Helsinki area and age- and gender matched controls were assigned to all type 2 diabetes cases. The case group is composed of 307 unrelated randomly selected individuals with type 2 diabetes (146 males and 161 females, mean age 61 (55–67) years, mean BMI 28.7 (26.0–31.7)). The control group consisted of 307 unrelated individuals with confirmed normal oral glucose tolerance and without a family history of diabetes (146 males and 161 females, mean age 60 (53–67) years, BMI 26.4 (24.1–29.2)).

### Extent of H2 haplotype linkage disequilibrium and genotyping of case-control and cardiovascular group populations

Using HapMap and the Human Genome assembly build 36.2, SNPs in or within 1000 base pairs (bp) upstream of the known genes located in the 3q24-25 region (P2Y12 locus) were identified. By means of DNA sequencing using BigDye v. 3.1 (Applied Biosystems, CA, USA) in 20 individuals, selected SNPs were probed for linkage disequilibrium with the known P2Y_12_ H2 haplotype SNPs using one of the P2Y_12_ H2 SNPs, rs2046934, as a marker of the P2Y12 H2 haplotype [11]. Single nucleotide polymorphisms (SNPs) that displayed high degrees of LD with the P2Y_12_ H1/H2 haplotype SNPs were selected for genotyping in a randomly selected sub-population of the DM case-control population (n = 295) using TaqMan or Sequenom and a haplotype map was constructed using the Haploview software [24].

Genotyping of the AMI (n = 3732) and DM (n = 614) case control populations and the cardiovascular group population (n = 6055) was performed using Sequenom (Sequenom Inc., CA, USA) or TaqMan ABI 7900 according to the manufacturers' instructions. Two different persons who were unaware of the phenotypic status of the study participants read all genotypes. For genotyping primers and probes, see table 2.

**Table 2:** Primers and probes used for genotyping of high linkage disequilibrium polymorphisms in P2Y_12_ and one in P2Y_13_.

SNP_ID	SNP location	Forward	Reverse	Mass Extension/Probes
rs1466684	P2Y_13_ Met-158-Thr	*5′-ACGTTGGATGGTGTTGCTTCCTTGTTGCTC-3′	*5′-ACGTTGGATGCGGTCTCAATCTTCATCTGG-3′	*5′-CATCTCCCTGCCAAATA-3′
rs1466684	P2Y_13_ Met-158-Thr	^#^5′-CGGTCTCAATCTTCATCTGGTTCTT-3′	^#^5′-CGATGGTGTTGCTTCCTTGTTG-3′	^#^5′-CTCAAGATCATATTTGG-3′
				^#^5′-CTCAAGATCGTATTTGG-3′
rs11922647	P2Y_12_ intron 1	*5′-ACGTTGGATGTTAAGGCATCCTTGTATCAC-3′	*5′-ACGTTGGATGCCCCTAACATATTTTTTGCCC-3′	*5′-CCTTCTGGTTTCAAAGTTAAA-3′
rs3821667	P2Y_12_ untranslated	*5′-ACGTTGGATGTGTTGATTCTGGAGGGTTTG-3′	*5′-ACGTTGGATGAGGAAAATACCAGATGCCAC	*5′-AGATGCCACTCTGCAGG-3′
rs2046934	P2Y_12_ intron 2	#5′-GCTATATGGCATCTACATCTTGGGAAT-3′	#5′-TGATTATTAAGAATATTTTATATAGAATCAATTTCACTTATCTCTGGTG-3′	#5′-TTGAAATGACATTTGTAATCT-3′
				#5′-AAATGACGTTTGTAATCT-3′

Some SNPs were genotyped with both Sequenom and TaqMan in different populations. *Sequenom, # TaqMan.

### Statistical analysis

In the AMI case control population, conditional logistic regression was used to calculate odds ratios and p values. The Cardiovascular group population was subjected to ANOVA and t-tests for continuous normally distributed variables, in case of non-normality Kruskal-Wallis test or Mann-Whitney test was used. Chi-2 test was used to test for significant differences in dichotomous variables. In the DM case control population, variables were log transformed for normal distribution. P-values were calculated using the GLM-ANCOVA using sex and age as covariates. Adjustment for multiple testing was not done. Statistical analyses were performed with SPSS.

### Power calculations

#### For myocardial infarction

Accepting a significance level of 0.05, 1244 AMI cases and 2488 controls have a power of 95% to detect a genotype relative risk of 1.20 for the P2Y_13_ Met-158-Thr polymorphism. Thus, it is unlikely that our result is a false negative finding.

#### For diabetes

Accepting a significance level of 0.05, 307 diabetes cases and 307 controls have a power of 35% to detect a genotype relative risk of 1.20 for the P2Y_13_ Met-158-Thr polymorphism. Accepting a significance level of 0.05, 532 diabetes cases and 5522 controls in the cardiovascular group population have a power of 79% to detect a genotype relative risk of 1.20 for the P2Y_13_ Met-158-Thr polymorphism. By analyzing both these two diabetes materials, it is unlikely that our result that the P2Y_13_ Met-158-Thr polymorphism is not associated with diabetes is a false negative finding. Powercalculations were performed using the program CaTS [25].

## Results

### Extent of P2Y_12_ H2 haplotype linkage disequilibrium

**Figure 1:**
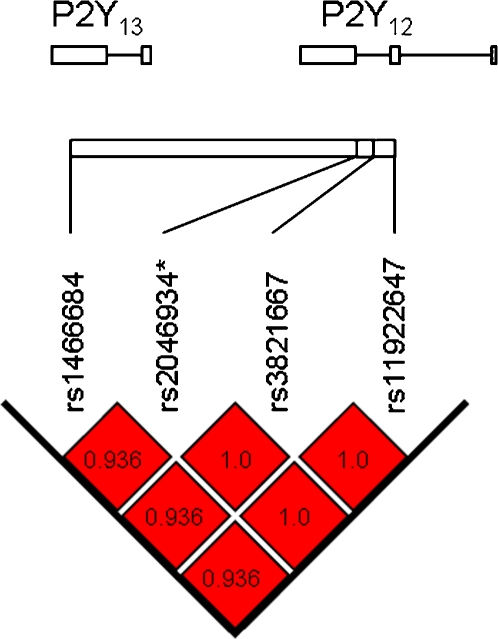
Linkage disequilibrium map of the P2Y12 H2/P2Y13 Thr-158 haplotype. All examined SNPs displayed a very high degree of linkage disequilibrium (D′ = 1.0; r^2^ = 0.936–1.0 (see figure for each individual r^2^ value).

The pilot linkage disequilibrium analysis of SNPs in the P2Y_12_ locus revealed three SNPs (rs1466684, rs38211667, rs11922647) that showed signs of LD with the known P2Y_12_ H2 SNPs. Genotyping in a larger population (n = 295) confirmed complete LD of the three SNPs with the P2Y_12_ H2 haplotype SNPs (D′ = 1.0, r^2^ = 0.936–1.0, figure 1) [11]. Two of the three SNPs were located in or in close proximity to the P2Y_12_ gene: rs38211667 in the non-coding region of P2Y_12_ exon 2 and rs11922647 within 1000 bp of transcription start of transcript variant 2 of the P2Y_12_ gene. The third SNP (rs1466684) was found to be a non-synonymous SNP of the P2Y_13_ gene, causing a Met-Thr amino acid substitution at position 158 of the P2Y_13_ receptor. The complete LD of the P2Y_12_ H2 haplotype with the P2Y_13_ Thr-158 variant thus defines a novel P2Y_12_ H2/P2Y_13_ Thr-158 haplotype. The P2Y_13_ Met-158-Thr polymorphism was selected as reference SNP for the P2Y_12_ H2/P2Y_13_ Thr-158 haplotype in subsequent genotyping.

### Genotyping of the P2Y_12_ H2/P2Y_13_ Thr-158 haplotype in the AMI and DM case control populations

In the AMI case control population, containing 3273 individuals, 92.8% of those eligible for the case–control study were genotyped successfully (table 3), representing 1134 pairs in total (n = 1134 cases and one to two control subjects matching every case (n = 2139). Genotype frequencies were in accordance with Hardy–Weinberg Equilibrium.

**Table 3:** Genotyping of Met-158-Thr in AMI cases and corresponding controls.

	Met-158-Met	Met-158-Thr	Thr-158-Thr	Met-158-Thr+Thr-158-Thr	Total
**Control count n (%)**	1437 (67.2)	638 (29.8)	64 (3)	702 (33)	2139 (100)
**AMI count n (%)**	772 (68.1)	332 (29.3)	30 (2.6)	362 (32)	1134 (100)
*Early onset AMI*					
**EO Control count n (%)**	715 (66)	323 (29.8)	31 (2.9)	354 (33.1)	1069 (100)
**EO AMI count n (%)**	368 (64.7)	176 (35.3)	16 (3.2)	192 (34.3)	560 (100)
*Late onset of AMI*					
**LO Control count n (%)**	722 (67.5)	315 (29.4)	33 (3.1)	348 (33)	1070 (100)
**LO AMI count n (%)**	404 (70.4)	156 (27.2)	14 (2.4)	170 (30)	574 (100)
*Family history of AMI*					
**FH Control count n(%)**	723 (68.3)	301 (28.4)	35 (3.3)	336 (32)	1059 (100)
**FH AMI count n (%)**	387 (69)	157 (28)	17 (3)	174 (31)	561 (100)
*No family history of AMI*					
**NFH Control count n(%)**	714 (66.1)	337 (31.2)	29 (2.7)	366 (34)	1080 (100)
**NFH AMI count n (%)**	385 (67.2)	175 (30.5)	13 (2.3)	188 (33)	573 (100)
*Early onset and family history of AMI*					
**EO+FH Control count n (%)**	373 (67.6)	159 (28.8)	20 (3.6)	179 (32)	552 (100)
**EO+FH AMI count n (%)**	197 (67.9)	87 (30)	6 (2.1)	93 (32)	290 (100)

The AMI case group (*n* = 1244) contains the early onset (EO), late onset (LO), family history (FH) and no family history (NFH) as well as early onset with family history (EO+FH) subgroups. *Both heterozygous and homozygous P2RY_13_ Thr-158 carriers.

No association of the P2Y_13_ Met-158-Thr polymorphism (Thr-158-Met and Thr-158-Thr vs. Met-158-Met) was found with AMI (OR = 0.96, 95% C.I. 0.82–1.12, P = 0.63). Also, no differences were seen in the AMI case subpopulations (EO OR = 1.06, 95% C.I. 0.85–1.31, P = 0.62; FH OR = 0.98, 95% C.I. 0.78–1.22, P = 0.83; EO+FH OR = 1.0, 95% C.I. 0.74–1.36, P = 1.0).

In the diabetes mellitus (DM) case control study 576 individuals (93.8%) were genotyped successfully. No associations of the P2Y13 polymorphism (Thr-158-Met and Thr-158-Thr vs. Met-158-Met) were found with diabetes mellitus or any examined DM risk factor (table 4).

**Table 4:** Genotyping of the P2Y_12_ H2/P2Y_13_ Thr-158 haplotype in a diabetes mellitus (DM) case control population (307 DM cases, 307 controls).

Controls (n = 289)§	Met-158 [n]#	Thr-158* [n]#	p-value
BMI (kg/m^2^)	26.4(24.1–29.2) [213]	26.6(24.2–29.3) [70]	**0.49**
WH	0.88(0.82–0.95) [211]	0.88(0.82–0.96)[70]	**0.64**
fP-Glu (mmol/L)	5.42(5.09–5.80) [214]	5.50(5.06–5.93) [73]	**0.56**
P-Glu [120 min] (mmol/L)	5.65(4.97–6.70) [191]	5.82(5.02–6.75) [64]	**0.90**
fP-INS (mU/L)	7.66(4.95–10.22) [210]	6.55(4.66–9.47) [68]	**0.10**
Triglycerids (mmol/L)	1.17(0.92–1.67) [198]	1.32(0.81–1.69) [65]	**0.86**
HDL-cholesterol (mmol/L)	1.36(1.16–1.66) [199]	1.37(1.18–1.73) [64]	**0.91**
HOMA	1.76(1.18–2.53) [207]	1.68(1.05–2.49) [68]	**0.13**

Diabetes mellitus (n = 287)§	Met-158 [n]#	Thr-158* [n]#	p-value
Age at onset (years)	53(46–59) [201]	53(48–60) [74]	**0.67**
BMI (kg/m^2^)	28.7(25.9–31.7) [206]	28.2(25.7–31.2) [78]	**0.53**
WH	0.93(0.87–0.99) [202]	0.94(0.90–1.00) [77]	**0.76**
fP-Glu (mmol/L)	9.49(7.90–11.75) [207]	8.87(7.12–11.35) [78]	**0.28**
P-Glu [120 min] (mmol/L)	15.43(11.67–20.18) [108]	14.24(12.37–16.84) [37]	**0.16**
fP-INS (mU/L)	11.95(6.99–21.15) [198]	11.54(7.87–19.09) [77]	**0.60**
Triglycerids (mmol/L)	1.57(1.15–2.21) [197]	1.66(1.21–2.39) [75]	**0.17**
HDL-cholesterol (mmol/L)	1.19(1.02–1.45) [191]	1.15(1.01–1.36) [71]	**0.31**
HOMA	4.98(3.00–9.20) [197]	4.70(3.00–9.88) [77]	**0.30**

Variables were log-transformed for normal distribution. P-values were calculated using the GLM-ANCOVA using sex and age as covariates. No associations were found with neither DM nor any known DM risk factor. *Both heterozygous and homozygous P2Y_12_ H2/P2Y_13_ Thr-158 carriers. §Genotyping failed in 20 cases and 18 controls. #median with interquartile range (25th–75th percentile), [number of observations].

### Genotyping of P2Y_13_ Met-158-Thr in the cardiovascular group population

5846 individuals (96.5%) of 6055 in the cardiovascular group (CVG) were genotyped successfully. No association was found for the P2Y_13_ Met-158-Thr polymorphism (Thr-158-Met and Thr-158-Thr vs. Met-158-Met) regarding any of the examined cardiovascular risk factors, including systolic blood pressure, diastolic blood pressure, BMI, waist circ., diabetes, total cholesterol, triglycerides, HDL, LDL, CRP, smoking or alcohol intake (table 5). Furthermore, no stronger association with the above mentioned risk factors was seen in the homozygous P2Y_13_ Thr-158 group compared to the heterozygous Met-158-Thr carriers (data not shown).

**Table 5:** Association of known cardiovascular risk factors with the P2Y_12_ H2/P2Y_13_ Thr-158 genotype in the cardiovascular group (CVG).

Cardiovascular risk factors	Met-158-Thr genotype¤	Mean±st dev	P value (two-tailed)
Systolic blood pressure (mm Hg)	Met	141.6±19.1	0.60
	Thr¤	141.3±19.1	
Diastolic blood pressure (mm Hg)	Met	87.3±9.5	0.15
	Thr¤	86.9±9.4	
Body-Mass Index (weight/kg×kg)	Met	26.0±4.0	0.16
	Thr¤	25.8±4.0	
Waist (cm)	Met	84.7±12.9	0.09
	Thr¤	84.1±13.0	
Diabetes mellitus (%)*	Met	8.8	0.94
	Thr¤	8.9	
Cholesterol (mmol/l)*	Met	6.2±1.0	0.93
	Thr¤	6.2±1.1	
Triglycerides (mmol/l)*	Met	1.4±0.9	0.38
	Thr¤	1.4±0.8	
HDL (mmol/l)*	Met	1.4±0.4	0.78
	Thr¤	1.4±0.4	
LDL (mmol/l)*	Met	4.2±1.0	0.96
	Thr¤	4.2±1.0	
LDL/HDL ratio*	Met	3.2±1.1	0.91
	Thr¤	3.2±1.2	
CRP (mg/L)*#	Met	§1.4 (0.7–2.8)	0.83
	Thr¤	§1.4 (0.7–2.9)	

Gaussian distribution was observed for all above risk factors except CRP that showed a natural logarithmic distribution. *n* = 6055. * *n* = 5540. § = median, interquartile range. ¤Thr = Both heterozygous and homozygous P2RY_13_ Thr-158 carriers.

## Discussion

In this study we show that the P2Y_12_ H2 haplotype [11] is in complete linkage disequilibrium with the non-synonymous Met-158-Thr polymorphism in the P2Y_13_ gene, defining a P2Y_12_ H2/P2Y_13_ Thr-158 haplotype. Based on the observed LD between the studied P2Y_12_ and P2Y_13_ polymorphisms, we assume that all disease and risk factor associations made with the P2Y_13_ Met-158-Thr polymorphisms are also valid for the P2Y_12_ H1/H2 haplotypes. We hypothesized that this finding could provide a potential mechanistic explanation to the previously observed clinical associations of the P2Y_12_ H2 haplotype with CAD, PAD or platelet function [11], [12], [14], since the receptors share the same ligand. However, no associations of the P2Y_13_ Met-158-Thr polymorphism with AMI or DM were found in our large material. Indeed, no associations with any of the investigated cardiovascular or diabetes mellitus risk factors were observed. This was unexpected, since strong associations have been reported between CAD, PAD, AMI and diabetes [18]–[20]. All studies involving the P2Y12 H2 haplotype are listed in table 6.

**Table 6:** Published studies on the P2Y12 H2 haplotype and platelet ADP response and cardiovascular disease.

Study author	Study population (n)	Reported outcome
Fontana [11]	98	P2Y12 H2 haplotype is associated with increased ADP-induced platelet aggregation
Fontana [11]	514	P2Y12 H2 haplotype is associated with peripheral arterial disease
Cavallari [14]	1378	The P2Y12 H2 haplotype is associated with coronary artery disease
Angiolillo [30]	119	The P2Y12 H2 haplotype does not influence platelet response to clopidogrel
Hetherington [31]	200	No association of P2Y12 H2 haplotype with ADP-induced platelet aggregation
von Beckerath [17]	416	P2Y12 gene H2 haplotype is not associated with increased adenosine diphosphate-induced platelet aggregation after initiation of clopidogrel therapy with a high loading dose
Schettert [16]	540	No asociation of P2Y12 H2 haplotype and an increased risk of cardiovascular events in a population with CAD
Amisten *et al*	10401	The P2Y12 H2/P2Y13 Thr-158 haplotype is not associated with AMI, cardiovascular risk factors or diabetes

The concentration of extracellular nucleotides in the blood is tightly regulated by ectonucleotidases on leukocyte and endothelial cells to prevent excessive ADP accumulation and subsequent platelet activation [26]. Red blood cells contain millimolar amounts of ATP and are therefore a major source of nucleotides in the blood [6], [27]. This ATP pool could potentially be an important contributor to the regulation of platelet activation. Recently, it was shown that extracellular ADP activates P2Y_13_ expressed on red blood cells, resulting in a subsequent decreased release of nucleotides from the red blood cells in a classic negative feedback manner [6]. It is possible that this negative feedback loop might be important in the regulation of nucleotide-induced platelet activation *in vivo*. Thus, a non-synonymous polymorphism leading to a structurally and functionally altered P2Y_13_ could potentially alter the nucleotide concentrations in the blood stream, thereby affecting platelet activation *in vivo*. Indeed, *in silico* analysis using the polymorphism phenotyping prediction software PolyPhen [28] indicated that the P2Y_13_ Met-158-Thr amino acid substitution could possible affect the function of the P2Y_13_ receptor.

In 2002 Fontana *et al* reported the P2Y_12_ H2 haplotype to be associated with a gain of function in terms of ADP induced platelet aggregation. The polymorphisms in the H2 haplotype are either located in intronic regions of the gene or were silent, i.e. causing no alterations of the P2Y_12_ receptor protein. The possibility remained that the polymorphisms could potentially be coupled to mRNA processing or translation events, thereby altering P2Y_12_ receptor protein expression. However, no such data has been presented.

In a subsequent study, Fontana *et al* also reported an association between the P2Y_12_ H2 haplotype and PAD [12]. PAD causes an increased atherosclerotic burden throughout the whole cardiovasculature and patients with PAD have a marked increase in coronary artery disease [29]. The progression of chronic atherosclerotic lesions is mainly driven by an inflammatory reaction, with recruitment of inflammatory cells and subsequent reactive changes in the vessel wall [21]. In acute thrombotic complications of atherosclerosis, such as myocardial infarction, platelets constitute the major role. A gain of function polymorphism leading to increased platelet reactivity would likely be more prominent when studied in a setting were platelet activation is a main pathogenic factor, such as AMI. However, to our surprise, genotyping in several thousand individuals revealed no association with AMI.

The early onset or family history AMI case-control subpopulations are believed to contain a stronger genetic component of AMI. The lack of association also in these populations emphasizes further that the P2Y_12_ H2/P2Y_13_ Thr-158 haplotype is not associated with cardiovascular disease.

It has been proposed that the P2Y_12_ H2 haplotype might be involved in the variation in response to clopidogrel treatment. However, subsequent studies have not been able to confirm that variations in response after a high loading dose of clopidogrel are associated with the haplotype. The failure to confirm this hypothesis agrees well with our study and supports the lack of association of the P2Y_12_ H2/P2Y_13_ Thr-158 haplotype with cardiovascular disease.

In conclusion, we found that the P2Y_13_ Met-158-Thr polymorphism was in complete LD with the P2Y_12_ H2 haplotype, defining a novel P2Y_12_ H2/P2Y_13_ Thr-158 haplotype. Genotyping of more than 10 000 individuals in three separate study populations revealed no associations with AMI, DM or related risk factors. Therefore, it seems very unlikely that the examined polymorphisms of the P2Y_12_ and P2Y_13_ genes contribute to the pathogenesis of cardiovascular disease or diabetes mellitus**.**
